# Intervertebral Disc Nucleus Repair: Hype or Hope?

**DOI:** 10.3390/ijms20153622

**Published:** 2019-07-24

**Authors:** Gauri Tendulkar, Tao Chen, Sabrina Ehnert, Hans-Peter Kaps, Andreas K Nüssler

**Affiliations:** Siegfried Weller Institute for Trauma Research at the BG Unfallklinik Tübingen, Eberhard Karls Universität Tübingen, Schnarrenbergstrasse 95, 72076 Tübingen, Germany

**Keywords:** intervertebral disc, nucleus pulposus, scaffold, replacement, repair

## Abstract

Chronic back pain is a common disability, which is often accredited to intervertebral disc degeneration. Gold standard interventions such as spinal fusion, which are mainly designed to mechanically seal the defect, frequently fail to restore the native biomechanics. Moreover, artificial implants have limited success as a repair strategy, as they do not alter the underlying disease and fail to promote tissue integration and subsequent native biomechanics. The reported high rates of spinal fusion and artificial disc implant failure have pushed intervertebral disc degeneration research in recent years towards repair strategies. Intervertebral disc repair utilizing principles of tissue engineering should theoretically be successful, overcoming the inadequacies of artificial implants. For instance, advances in the development of scaffolds aided with cells and growth factors have opened up new possibilities for repair strategies. However, none has reached the stage of clinical trials in humans. In this review, we describe the hitches encountered in the musculoskeletal field and summarize recent advances in designing tissue-engineered constructs for promoting nucleus pulposus repair. Additionally, the review focuses on the effect of biomaterial aided with cells and growth factors on achieving effective functional reparative potency, highlighting the ways to enhance the efficacy of these treatments.

## 1. Introduction

Degenerative disc disease (DDD) is a common clinical condition that causes chronic back pain [1]. Lower back pain is one of the leading causes of disability and thus places a high burden on healthcare systems worldwide; yet, it is not among the top 10disorders receiving research funding [2]. Clinical problems associated with intervertebral disc degeneration, including disc biology, disease pathophysiology, or biomechanics, have received close attention, which has enhanced the basic understanding of these issues [3,4,5,6,7]. Furthermore, advanced novel research mainly focuses on the fundamental topic of replacement and repair of the damaged tissue, which has accelerated its clinical translation [1,8,9,10,11,12]. The objective of this review is to highlight the need for therapeutic alternatives that allow repair with replacement of intervertebral discs (IVDs). Research that has focused on repair with replacement of the nucleus pulposus (NP) is discussed.

## 2. IVD Degeneration

IVDs are cartilaginous structures between the vertebral bodies that mainly provide flexibility and elasticity [13] and have a wide range of movement to the spine as a whole. In addition, IVDs strongly provide pressure and tensile resistance while transmitting mechanical load through the spinal column [14] and therefore support a variety of loads during daily activities. A healthy IVD is comprised of a proteoglycan-rich gelatinous center called the NP, which is peripherally enclosed by the collagen-rich annulus fibrosus (AF) and the cartilaginous endplate (CEP), which limit the peripheral rim of the disc superiorly and inferiorly [15]. The primary components of IVDs are water, cells (mainly chondrocyte-like cells and fibroblasts), proteoglycan, collagen, and other matrix components [16]. Fibrillar collagens, aggrecan, and water are the three main structural components of the IVD, all together contributing to around 90–95% of the volume of a healthy IVD [17], although their percentages vary across the disc [14]. 

**Figure 1:**
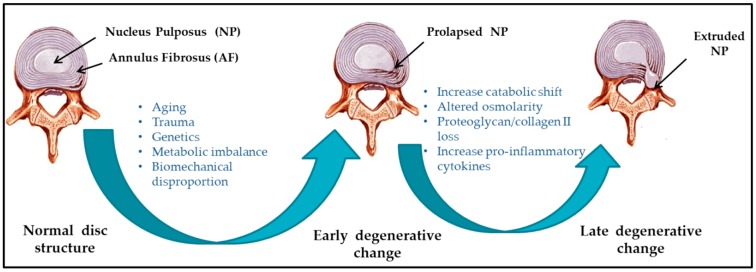
Schematic illustration of intervertebral disc (IVD) pathophysiology during degeneration.

Several etiological factors such as aging, smoking, infection, abnormal biomechanical loading, and nutrition insufficiency are thought to be involved in the pathogenesis of IVD degeneration [17,18]. Among these factors, genetic heritability is estimated to account for up to 74% [19]. As the degeneration process is highly correlated with aging, its pathologic changes occur starting from the second decade of life [6,20]. Substantial changes in biochemical composition and progressive loss of structural integrity are hallmarks of IVD degeneration [15] (as illustrated in Figure 1, where curved arrows define the transition from normal disc structure to later degenerative disc), which occurs mostly in adults aged over 30 years in one or more discs or during trauma and injury. Loss of proteoglycans and a decrease in the ratio of proteoglycan to collagen [17] consequently results in the loss of hydrostatic properties, which induces structural wear of the IVD [21] and thus progresses towards a fibrotic nature. Dehydration of NP and gradual disappearance of the NP–AF border contributes to the loss of normal architecture. Stress distribution over the NP tends to reduce at the center and accumulates more pressure around the periphery, effectively disabling the NP’s load transfer function [22]. Due to a lack of intradiscal pressure, the load absorption and transmission in such dehydrated discs is significantly altered and subsequently, it results in disc-height reduction, osteophyte formation, facet joint arthritis, and deformation of vertebral bodies [23]. With continuing degeneration, the structural deficit is accompanied by leakage of the central NP material through cracks in the AF into the periphery. This results in immune cell activation, thereby evoking chronic back pain [24,25]. Since biochemical changes within IVDs have not yet been directly associated with chronic back pain, it is difficult to determine if the observed changes are due to aging or pathology [26]. 

IVD degeneration often results in lower back pain but is not always the only causative factor. Location of the affected disc, degree of nerve damage, and amount of pressure on the spinal column contribute to define the degree of degeneration. For example, some patients may not feel pain, while others with similar degrees and extents of IVD damage may experience chronic back pain. Therefore, the degree and extent of degeneration does not correlate with the degree of pain. IVD degeneration is the most common cause of lower back pain [27]. The worldwide prevalence of chronic back pain is approximate 60%, with the majority seen in the elderly [28].

## 3. NP Replacement

**Figure 2:**
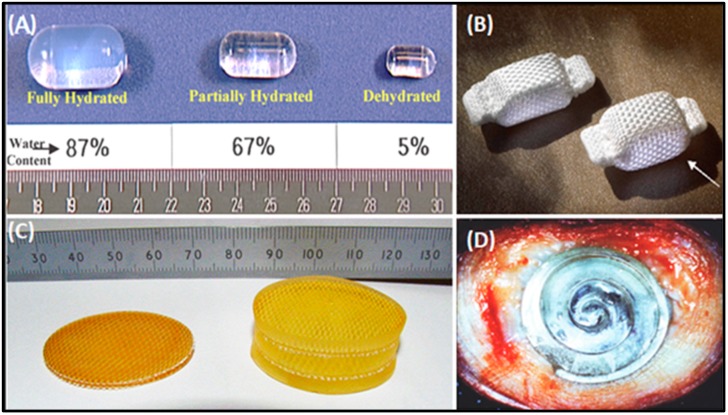
Nucleus replacement devices. (**A**) The Aquerelle poly (vinyl alcohol) hydrogel has a swelling pressure similar to the nucleus pulposus (NP) in vivo. Once implanted, its final volume depends on the water content at equilibrium (reprinted with permission from Stryker Spine, Allendale, NJ, USA). (**B**) The Prosthetic disc nucleus (PDN-SOLO) device in dehydrated (left) and hydrated (right; as indicated by arrow) states. This device was designed to swell both in height and width within the disc space. The porous polyethylene weave allows fluid to pass into the hydrophilic core, which causes the device to expand vertically and horizontally. This process maximizes the device’s footprint on the vertebral endplates (reprinted with permission from Raymedica Inc., Minneapolis, MN, USA). (**C**) The Neudisc hydrogel, pre-hydration (left) and post-hydration (right). Hydration occurs in an anisotropic fashion, mainly in the vertical plane (reprinted with permission from Replication Medical, Inc., New Brunswick, NJ, USA). (**D**) The Newcleus Spiral Implant; once implanted, the device reconstitutes its original spiral shape. It localizes in place of the nucleus pulposus, which reconstitutes the volume, sparing the annular fibers (reprinted with permission from Zimmer Spine, Warsaw, IN, USA) [10,45].

Current surgical intervention aims to alleviate the symptoms instead of providing a complete cure [29,30,31,32,33,34]. Moreover, the avascular nature and low cellularity of IVDs often limit the regeneration potential [5,35,36,37,38]. A number of biomaterials/implants of good quality that can mimic NP tissue have been developed (refer Figure 2) and investigated in vitro and ex vivo [39,40]. NP replacement strategies administer a biocompatible material to retain the native biomechanics and to promote tissue integration [10,41,42]. In situ hydrating synthetic polymers (e.g., copolymeric hydrogel encased in a polyethylene fiber jacket polyacrylonitrile and polyacrylamide (PDN™)) for the NP approach have the longest history of clinical use. In situ forming synthetic polymers (e.g., chemically crosslinked biomaterial NuCore™, BioDisc™) represent another class for NP replacement. Controlling the degree of swelling remains one of the main advantages of synthetic polymers, which are otherwise used to mimic native disc properties [43]. However, excessive implant stiffness, endplate overloading and fracture, and fragmentation of gel upon swelling [44] are major drawbacks. Moreover, in vivo and clinical tests of mechanical repair have failed to promote tissue integration and restore the native biomechanics of the spine as a whole [10]. For these reasons, the use of biomaterials for NP repair has recently been approached with caution.

NP injuries following annular injuries highlight the need for long-term stable repair strategies that yield native disc function through containment of the NP within the IVD and regeneration of native biomechanics. The exclusive focus on the mechanical repair of NP is the prime suspect of the failure of NP repair strategies [10]. In order to overcome the inadequacies of current mechanical implants of the nucleus pulposus, there is a definite need to enhance long-term regeneration to achieve persistent repair, thereby preventing further degeneration [46]. Utilizing the principle of tissue engineering, in combination with biomaterials, cells and/or growth factors potentially aim to regain the functional potency of intervertebral discs [28,47,48,49,50]. So far, common approaches for NP repair have involved the use of natural and synthetic biomaterials in combination with primary disc cells, stem cells, or growth factors [51]. Although the initial screening and characterization of novel repair strategies is valuable, it is critical for their continued progress to assess their safety and efficacy in vivo or ex vivo. In vivo models that mimic human IVD pathophysiology and biomechanics would be a gold standard for such assessments. In this review, we discuss biological NP repair strategies that have been or can be implemented in preclinical in vivo models and the remaining scientific challenges of successful NP repair.

## 4. NP Repair

Tissue engineering approaches over the past few years have been addressing the objective to restore functional and structural features of the healthy IVD. Reparative treatment mainly targets intervention at early stages of IVD degeneration to restore extracellular matrix (ECM) homeostasis, control inflammation, and prevent angiogenesis [2]. Current surgical procedures mainly focus on alleviating symptoms associated with IVD degeneration but fail to promote tissue remodeling. Tissue engineering offers an alternative to design biomaterials by encompassing cells and growth factors that will aid IVD tissue regeneration. Thereby, it offers multiple strategies to prevent and possibly cure IVD degeneration by encouraging disc repair. The exact mechanisms of IVD regeneration are still not known, however several studies have focused on the effect of segmental distraction in IVD disease [11,12,52]. Synthetic and / or natural material based scaffolds for IVD tissue engineering were regarded as the prominent method over the past decades [53,54]. In spite of considerable progress, some issues related to scaffold integration and tissue repair still remained unsolved [55]. Alternatively, scaffold free tissue engineering (refer Table 1) is an emerging field, where cells, growth factors, or peptide delivery are mainly responsible for regaining the tissue integrity upon the application [56,57]. Recently, stimulatory factors together with cells either unaided or together with biomaterials have aimed to provide suitable repair site to ensure maximum cell differentiation or deposition of appropriate ECM. Nonetheless, selection of biomaterials, cells and appropriate stimulatory factors is crucial as the ideal combination is yet to be established.

## 5. Growth Factors

Growth factors, such as bone morphogenetic proteins (BMPs), members of the transforming growth factor beta (TGF- β) super family, have shown an effective role in promoting cell proliferation in vitro as well as in vivo [11,58,59]. BMP2 treatment enhanced matrix production e.g., collagen II and aggrecan in rat, rabbit, and human IVD cells in vitro [60,61,62]. Interestingly, this effect was more distinct in adult rabbit IVD cells than in adolescent rabbit IVD cells [63]. In vivo (rabbit annular puncture model) intradiscal BMP2 injection did not impede progression of injury-induced degeneration as shown by Kong et al. [64]. In addition, Huang et al. showed BMP2 injection provoked acceleration of IVD degeneration and osteogenic responses near the vertebral endplates [65]. Regenerative effects of BMP2 on degenerated IVDs in vivo are thus controversial. BMP7 (osteogenic protein-1), another growth factor, is widely studied for NP regeneration. BMP7 had shown positive effects on ECM production [66,67,68]. An in vivo rabbit model showed disc height restoration and an increase in proteoglycan content upon recombinant human (rh) BMP-7 treatment [69,70]. Furthermore, in a rat IVD compression model its anti-catabolic effects were confirmed [71]. However, recently Willems et al. [72] and Van Dijk et al. [73] demonstrated that intradiscal application of rhBMP7 did not induce regeneration in a canine model of spontaneous intervertebral disc degeneration (IDD) and human derived NP tissue, respectively. Additionally, growth and differentiation factor 5 (GDF-5; also known as BMP-14) has shown positive impact on NP regeneration in vivo [74,75]. Thus, clinical trials of single intradiscal injection of rhGDF-5 and their resultant data with respect to adverse effect development and neurological status of patients have been published (http://clinicaltrials.gov/show/: NCT01182337; NCT01158924; NCT00813813; NCT01124006) [76]. Apart from this, a need for sustained delivery of TGF-β during regeneration has been proposed from a mouse IVD compression model, where TGF-β showed AF cell proliferation with increased gene expression of aggrecan and collagen II [77]. Moreover, insulin-like growth factor-1 (IGF-1) [78,79], basic fibroblast growth factor (bFGF) [79,80], epidermal growth factor (EGF), and platelet derived growth factor (PDGF) [79], as well as platelet-rich plasma (PRP) [81] have been reported to enhance IVD cell proliferation and matrix synthesis. In vivo studies (rabbit model of IDD induced by annular puncture) on GDF-5 injection showed restorative effect on disc height with improved histologic and magnetic resonance imaging (MRI) (showing improved hydrophilic properties of NP) findings [74]. Also, synergistic effects of multiple growth factor cocktails can be considered as therapeutic approaches [82]. In parallel, the effects of growth factors on human degenerative discs deserve further investigations. Despite reasonable success achieved by growth factors, some important limitations such as dose, delivery, half-life, side effects etc. should be contemplated in the application of growth factor therapy. Moreover, the effects of single dosage/injections of growth factors are not sustainable over a longer period, while multiple dosages may cause inflammatory reactions. In addition, injections of TGF-β, IGF-1 and bFGF may induce unwanted angiogenesis that may accelerate further deterioration [75]. It may therefore limit its clinical application. Nevertheless, further studies are required to confirm the safety and efficacy of intradiscal application [83]. Moreover, the injected growth factor does not distort the structural integrity and thus, biomechanical alteration even after the growth factor treatment may fail in clinical settings [84,85]. To overcome the demerits of growth factor delivery, gene therapy as an alternative strategy has been investigated to retard IDD. However, their effects and safety issues on human disc are not clear yet. 

## 6. Cell-Based Therapies

Cell based therapy for IVD nucleus repair mainly aims for NP like cells injections, addressing the imbalance of biochemical environment (proteoglycan synthesis and water content). Stimulation of the residing cells is insufficient to achieve tissue repair; hence, diseased phenotype properties of the native NP cells certainly limit their use. Thus, injecting functional cells aimed to overcome this problem by compensating cell death and disc shrinkage [51,86]. Concomitantly, Abbott et al. proposed NP cells do possess regeneration potential even in severe status of degeneration [87]. Several cell-based strategies have been investigated to retard NP degeneration, using different IVD model system [88,89,90,91], with NP and/or AF cells, articular chondrocytes, or mesenchymal stem/stromal cells (MSCs). Feng et al. summarized GDF-5 a suitable growth factor for inducing NP-like cells based on its positive effect on the differentiation of MSCs towards an NP-like phenotype [92]. The effect of growth factors such as TGF-β, IGF-1, FGF-2, PDGF and GDF-5 on the differentiation of MSCs into NP-like cells have been investigated [92]. Fundamentally, the transplanted MSCs are expected to differentiate, maintain and enhance the function of existing NP cells to reverse the IDD. Thus, preclinical studies will be required to confirm the functional potency of the MSC-based therapy for IDD. In the Euro Disc study, percutaneous injection of autologous IVD cells demonstrated disc height restoration and pain reduction for up to two years following transplantation [93,94]. However a lack of placebo-controls is the drawback of the results. Similarly, in the NuQu phase I safety study percutaneous injections of allogeneic juvenile knee chondrocytes into degenerated lumbar IVDs of 15 human patients showed improved pain scores and radiological parameters within a period of 12 months of observation. Therefore, a phase II clinical trial regarding safety and efficacy of allogenic chondrocytes injection is ongoing [94]. Furthermore, several phase I and II studies with autologous and allogeneic MSCs are currently being performed (NCT01290367; NCT02037204; NCT02338271; NCT01860417) [94]. Good accessibility and the ability of MSCs (bone marrow, adipose and synovial tissue derived) to differentiate into different cell types including chondrocyte like cells, promoted MSCs as prime source for cell therapies for several diseases and in IVD regenerative treatment [95,96]. Intradiscal delivery of bone marrow and adipose derived MSCs has demonstrated to promote regeneration by maintaining cell viability and proliferation, obtaining IVD-like phenotypes, and providing expression of typical chondrocyte markers, in several studies using rabbit, rat, dog, and goat models [9,97,98,99,100]. Nevertheless, one common problem affecting the healing satisfaction is the tendency of hypertrophic differentiation of MSCs [101,102]. How to achieve an ideal IVD repair mainly relies on manipulation of hypertrophic chondrogenesis of the injected and/or implanted MSCs. 

In parallel, the use of notochordal cells is also being considered, no matter from allogenic, autologous or xenogenic origin [103,104,105]. Risbud et al. has previously proposed that morphology and size variation correlates to different stages of maturation and/or function of NP cells derived from notochordal precursors [106]. Moreover, it has been speculated that degeneration is due to the selective loss of the notochordal cells fraction while considering overall reduction in the structural and functional activity of IVD cells. While there is considerable agreement, Bach et al. demonstrated the species (human, canine and porcine) specific regenerative effects of notochordal cell conditioned medium on human NP cells. It further confirmed the canine and porcine secreted factors exerted regenerative effect on human NP cells [107]. The fact that NP cells with diseased phenotype possess regeneration potential, allows autologous NP cells to be expanded using conditioned medium that these cells can be used as a source for cell therapy during nucleus repair. Likewise, stem cells can be differentiated towards NP like cell type. van Uden et al. has already addressed [48] the importance of hypoxia during NP repair. This key factor may restrict the success of the stem cell based therapy, as stem cells tend to die due to lack of oxygen. Serigano et al. demonstrated the effect of cell number on mesenchymal stem cell transplantation in canine disc degeneration model, where they showed high possibility of apoptosis, low cell viability, while maintaining microenvironment during stem cell transplantation [108]. Altogether, it affected the regeneration capability. However, in order to advance clinical translation of cell therapy, assessment of in vivo integration (in terms of functional and mechanical repair) in large animals is necessary. Therefore, a comparative analysis of cell types and sources of cells using large animal models is essential to enlighten the suitable strategy.

## 7. Injectable Hydrogels

Synthetic or biologically based injectable materials are largely focused on NP replacement, to stabilize and restore the function and structure of the discs and AF. Moreover, the biomaterial must satisfy the biomechanical strength with no migration and displacement, and durability with high wear resistance provoking low immune response. Injectable hydrogel over the solid-state scaffold opens up novel approaches in musculoskeletal application. Hydrogels mainly composed of natural polysaccharides (chitosan, chondroitin sulfate, hyaluronic acid), proteins (silk, resilin), or synthetic polymers (polyvinyl alcohol (PVA), polyacrylic acid, acrylamide), are emergent matrix substitutes in cartilage and IVD regeneration [109]. They are hydrophilic in nature, and have a water retention capacity between 20% and 99% by weight when placed in aqueous conditions. Therefore, these water soluble polymers are often used to build scaffolds by three-dimensional crosslinking either by covalent or physical methods. Hydrogels (depending on the physical structure and chemical composition) that mimic the mechanical stability and matrix composition of native IVD ECM, are a potential promising choice for IVD repair. Cell-free hydrogels and cell-seeded hydrogels are the two broad subtypes of injectable scaffolds found in literature [110,111].

Theoretically, biodegradability of some of the scaffolds allows remodeling of the scaffold in the regeneration process, while other scaffolds mechanically support and resist the compressive load for longer duration. For example, alginate and lyophilized chitosan gelatin scaffolds showed cytocompatibility towards the NP like cells, supporting cell growth. Li et al. stimulated NP cells using BMP-2 and BMP-7 heterodimer combined with fibrinhyaluronan hydrogel [112]. Their in vitro as well as ex vivo study demonstrated to produce aggrecan and collagen II by NP cells upon delivery, simulating in vivo conditions [112]. Moreover, cell numbers were found to be increased in alginate compare to lyophilized chitosan-gelatin based scaffolds, after 21 days of cell culture [51]. Hydrogels on the other hand resemble NP material, mainly due to their resilient and hydrophilic properties. Recently, several studies [91,110,111,113,114] have investigated the combination of cells and hydrogels to catalyze the tissue repair. Homogenous distribution of cells within defect size prior to gelation tends to support tissue repair [51]. To allow the sustainable effect of the growth factor Yan et al. administered the injection of poly (lactic-co-glycolic acid) (PLGA) microspheres loaded with recombinant human GDF-5 into a rat caudal disc degeneration model induced by needle puncture [115]. Sustained release of active GDF-5 for more than 42 days confirmed its therapeutic efficacy. Further Frith et al. [114] conducted a study examining the composite of injectable hydrogels (polyethylene glycol, hyaluronic acid, and pentosanpolysulphate) coupled with MSCs. In vivo examination of this scaffold composite together MSCs in rats, certainly supported the cartilage like tissue formation, thereby confirming the deposition of collagen II. However, there is a need to identify relevant combination of biomolecules and hydrogel material that may direct NP cell survival *in vivo,* in the challenging mechanical and physical microenvironment of the NP. While doing so, the biomaterial should withstand the mechanical load which ultimately contribute to the longevity of the scaffold.

## 8. Future Research Areas: Attempts to Regenerate the NP during Replacement 

**Figure 3:**
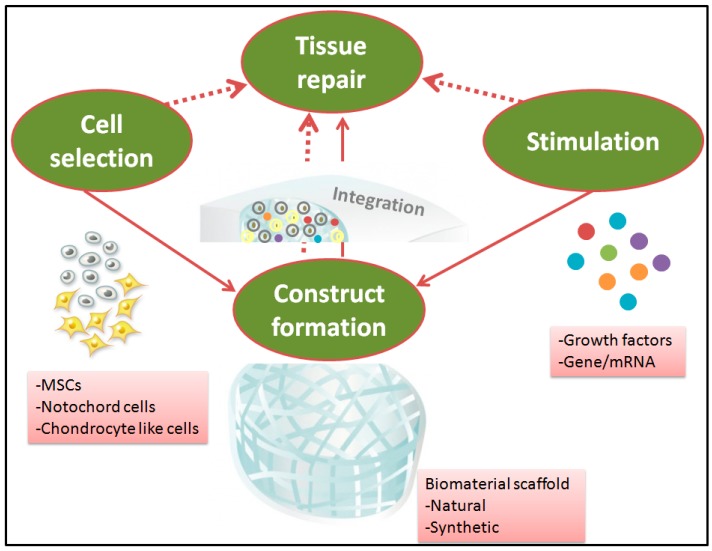
Synergetic tissue engineering strategies for nucleus pulposus repair.

The pathophysiology of the disc at different stages of degeneration is not well characterized yet. High complexity and heterogeneity of IVDs, mainly limits the understanding of IDD process. Therefore, NP repair requires comprehensive understanding of the degrees and the extents of IVD degeneration, followed by designing a scaffold with suitable structure and biological activity that will create conductive microenvironment for the biological interaction in vivo. Pre-requisite of the pivot joint (IVD) replacement often highlights the load-bearing factor, and therefore, mechanical replacement remains the conventional choice for severe degrees of IVD degeneration. However, IVD degeneration treatment highly demands for tissue repair aiming to generate functional neo-tissue formation (refer Figure 3, where red dotted arrows indicate the possible ways of tissue repair), while considering in vivo mechanical stimuli. Despite the fact that the biomechanical replacement offers a promising perspective for NP regeneration, comprehensive consideration of interdisciplinary strategies addressing biological and mechanical needs for NP repair certainly may achieve regeneration.

Tissue engineering strategies therefore should be directed to use scaffolds, cells and bioactive stimuli all together, as a reparative approach (refer Figure 3, where red solid arrows indicate the possible ways of combination), allowing cellular interaction for load-bearing application-targeted scaffolds. The impact of mechanical loading on cell behavior is one of the major considerations in this type of strategy, as scaffold’s long-term durability is expected not to cause further complication. Therefore, a precise material selection for NP repair that supports minimally invasive implantation, in situ fixation and defect filling are likely to have a higher success rate. In this scenario, an emergence of third generation biomaterials in the healthcare system is contributing to develop strategies of combining appropriate stimulatory factors/growth factors together with biomaterial and/or cells to enhance the endogenous regeneration potential [1,86], thereby assuring a potential tissue repair. Although, tissue engineering and regenerative medicine strategies aim at catalyzing an efficient restoration of single tissues (AF or NP), their short term success and unclear long term biological consequences mainly limit their clinical application. Therefore, restoration of two dissimilar tissues, the AF and NP collectively should be in focus. Recently, Moriguchi et al. demonstrated a tissue engineered IVD-construct: AF cells seeded on a collagen based hydrogel and NP cells seeded on an alginate hydrogel were formed into a single disc unit using preformed canine spine geometry molds [116]. Histological analysis confirmed host tissue integration over 16 weeks without elevation of immune reaction. Such strategies will have to be dealt with developing a scaffold that leads to regeneration of NP-AF concurrently. With rapidly accumulating knowledge on the advanced drug delivery, cell therapy and stimulatory agents, a more robust approach exploring the diverse biomaterials may provide superior efficacy-targeting NP repair and thus, advanced IVD degeneration therapy may work successfully [117,118]. Importantly, the experimental outcome should intend to correlate with the final aim of chronic back pain relief in humans while considering the clinical translation.

## 9. Conclusions

In the past decade, biomaterials either unaided or together with cells/growth factors have gained significant attention in the field of IVD regeneration. Here, we discussed NP repair strategies, where injectable hydrogels, cell and growth factor based therapies have been extensively studied both in vitro and in vivo. However, the conflict of tissue integration and load-bearing capacity limits so far a long-term application of currently available and well-tested NP repair strategies. Emergence of third generation biomaterials in the health care system, aided together with cells and growth factors is still more a fiction than a fact. Yet, it is our hope that the tissue engineering based approaches for NP repair will stimulate further research to seek more satisfying solutions to be added into the biomedical pipeline.

## Figures and Tables

**Table 1:** Advantages and disadvantages of scaffold-free IVD tissue engineering.

Methods	Categories	Advantages	Disadvantages/Limitations	References
Cell therapy	NP cells	No immune resistance Restricted to chondrogenic lineage	Donor-site morbidity Dedifferentiation issue Low proliferation ability Multiple surgical procedures	[88,89,90,91]
	MSCs	Abundant cell resources High proliferation rate and chondrogenic differentiation capacity Immunomodulatory abilities Simplicity and ease of the injection	Not restricted to chondrogenic lineage Potential disease transmission Tumorigenesis risk	[94,95,96,97,98,99,100,101,102]
Growth factors	TGF-β	Enhances cartilage formation and extracellular matrix production	No immediate structural and biomechanical alteration Biodegradation in vivo	[58,77]
	BMP2	Enhances ECM production and phenotypic characteristics of NP cells	Induces apoptosis, Col I accumulation, and aggrecan-production hindrance	[60,61,62,63,64,65]
	BMP7	Promotes proliferation and accelerates chondrogenesis	Short half-life time and biodegradation in vivo	[66,67,68,69,70,71,72,73]
	GDF-5	Induces NP-like differentiation of MSCs	Possible association between GDF-5 gene polymorphisms and IDD	[74,75,76]
	IGF-1	Enhances the ECM production and proliferation of IVD cells	Enhances glucose consumption and lactate concentration	[78,79]
Injectable hydrogel	Cell-free hydrogel	Physiological swelling and greasing	Limited payload	[51,110]
	Cell-seeded hydrogel	Active matrix synthesis Preserves the behavior of seeded cells	No direct cell contact	[111,112,113,114]

MSC: mesenchymal stem cell; BMP: bone morphogenetic protein; GDF: growth and differentiation factor; IGF: insulin-like growth factor; ECM: extracellular matrix; IDD: intervertebral disc degeneration.

## Abbreviations

AF	annulus fibrosus
BMP	bone morphogenetic protein
CEP	cartilaginous endplate
DDD	degenerative disc disease
ECM	extracellular matrix
GDF	growth and differentiation factor
IVD	intervertebral disc
IDD	intervertebral disc degeneration
MMP	matrix metalloproteinase
MSC	mesenchymal stem cells
NP	nucleus pulposus
